# Evaluation of a rapid device for serological in-clinic diagnosis of canine angiostrongylosis

**DOI:** 10.1186/1756-3305-7-72

**Published:** 2014-02-18

**Authors:** Manuela Schnyder, Kathrina Stebler, Torsten J Naucke, Susanne Lorentz, Peter Deplazes

**Affiliations:** 1 Institute of Parasitology, Vetsuisse Faculty, Winterthurerstrasse 266a, Zürich 8057, Switzerland; 2 Department of Zoology, Division of Parasitology, University of Hohenheim, Stuttgart, Germany; 3 Laboklin GmbH & Co. KG, Bad Kissingen, Germany; 4 Parasitus Ex e.V, Vollbergstrasse 37, Niederkassel 53859, Germany

**Keywords:** *Angiostrongylus vasorum*, Dogs, Serological diagnosis, Rapid device test, Antigen detection, Specificity, Sensitivity

## Abstract

**Background:**

*Angiostrongylus vasorum* is a potentially fatal canine nematode. Due to the high variability of clinical signs and the often chronic and subtle course of the infections, the diagnosis is particularly challenging. A rapid in-clinic assay (Angio Detect™ Test, IDEXX Laboratories, Westbrook, Maine, USA) for the serological detection of circulating antigen and intended for routine in-clinic diagnosis has been evaluated.

**Methods:**

Sensitivity was calculated with sera from 39 naturally infected dogs confirmed by Baermann-Wetzel analysis, while sera of 38 experimentally infected dogs were used for follow-up analyses, of which 10 were treated with imidacloprid/moxidectin. Cross-reactivity was tested with a total of 123 samples from dogs with proven parasitic infections with *Toxocara canis* (n = 21), *Ancylostoma caninum* (n = 4), *Crenosoma vulpis* (n = 18), *Oslerus osleri* (n = 3), *Eucoleus aerophilus*, (n = 6), *Dirofilaria immitis* (n = 28), *Dirofilaria repens* (n = 20), *Acantocheilonema reconditum* (n = 10) or *Dipetalonema dracunculoides* (n = 10) or multiple infections (n = 3). All sera were tested with the Angio Detect™ Test and with an ELISA for detection of circulating antigen of *A. vasorum*.

**Results:**

The sensitivity of the Angio Detect™ Test was 84.6% (95% C.I. 69.5 - 94.1%), while specificity was 100% (95% C.I. 97.6 - 100%). The sensitivity of the ELISA (94.9%, 95% C.I. 82.7 – 99.3%) was comparable with previous evaluations. In experimentally infected dogs, earliest positive results with the Angio Detect™ Test were observed 9 weeks post inoculation and 5 weeks later all sera were Angio Detect™ Test positive. After anthelmintic treatment, seropositive dogs turned negative again within 3 to 7 weeks after treatment. The evaluation of the colour intensity of the test strips confirmed the delay of approximately 3-4 weeks for antigen detection by the Angio Detect™ Test compared to the ELISA and its correlation with the time after infection.

**Conclusions:**

This study provided evidence of a good sensitivity and a very high specificity of the rapid device Angio Detect™ Test for detection of circulating *A. vasorum* antigen in dogs with suspected canine angiostrongylosis, representing a very simple and useful tool to be broadly applied in veterinary practices. The rapid detection of infected dogs is a key point for initiating an indispensable and urgent therapy.

## Background

Canine angiostrongylosis is caused by the metastrongylid *Angiostrongylus vasorum*, manifesting mainly in respiratory signs, coagulopathies and neurological signs [1], and can be fatal if left untreated [2-4]. Due to this high variability and the often chronic and subtle course of the infections, the diagnosis of this disease is particularly challenging. However, early and correct diagnosis are of paramount importance to prevent the onset of severe pathological changes, considering that they can be present even in the absence of clinical signs [5]. Adult stages of *A. vasorum* are localized in the pulmonary artery and the right heart of dogs, foxes and some other carnivores, while snails and slugs act as intermediate hosts [6]. The currently most employed method for diagnosis of *A. vasorum* in dogs is the isolation of first stage larvae excreted with faeces applying larval migration techniques such as the Baermann-Wetzel method, followed by microscopic identification of the larvae [7]. However, during prepatency or in case of low worm burdens with potential intermittent larval excretion [5,8] or when analysing posted faecal samples that arrive with delay at the laboratory and therefore contain inactive or dead larvae, the test may be negative. Also, differentiation from other lung worm larvae such as *Crenosoma vulpis* and *Filaroides* spp. or larvae that may be present in delayed samples (i.e. of *Strongyloides* and/or hookworms) or in samples collected from the ground (containing free-living or plant parasitic nematodes) needs experienced staff, in order to avoid misdiagnosing. This latter consideration is valid also for FLOTAC, a technique based on the counting of larvae in chambers after spinning faecal samples onto a surface and suggested to improve diagnosis of *A. vasorum* especially when larval migration techniques cannot be used [9].

Attempts for the development of serological methods for detection of *A. vasorum* – infections in dogs started already in 1971 with the detection of specific antibodies against *A. vasorum*[10], and were succeeded by follow-up studies in experimentally infected dogs [11,12]. The identification of immunogenic antigens of different molecular weight in crude extracts of adult female worms in the humoral response of infected dogs [13] was followed by the identification of stage-specific antigens also based on sera of dogs experimentally infected with *A. vasorum*[14]. In a consequent approach, Verzberger-Epshtein *et al*. [15] used polyclonal rabbit antiserum directed against whole adult worms for the detection of circulating *A. vasorum* antigen. Cross-reactions against several helminths and in particular against *C. vulpis*, a lung worm known to be widely distributed, were evaluated in a sandwich-ELISA, showing high specificity and sensitivity. However, samples from animals infected with *Dirofilaria immitis*, another nematode residing in the heart of definitive hosts in the adult stage and known to produce circulating antigens [16] were not investigated. These both potentially fatal canine nematodes are present in overlapping endemic areas in Europe, and this has to be accounted for when applying diagnostic methods in suspect cases or when analysing dogs in non-endemic areas with travel history. In fact, cross-reactions of sera from dogs infected with *A. vasorum* were detected using commercially available *D. immitis* test kits [17], confirming that antigens of *A. vasorum* and *D. immitis* may share epitopes responsible for potential cross-reactions in antigen detection tests. This fact has been considered during the development of another ELISA developed for the detection of circulating antigen of *A. vasorum* applying a monoclonal antibody which did not react with *D. immitis* excretory/secretory (E/S) antigen [18]. The use of this species-specific monoclonal antibody with polyclonal rabbit antibodies directed against *A. vasorum* adult E/S antigen allowed the detection of circulating antigens with a high specificity (94.0%) and sensitivity (95.7%). In further sandwich-ELISAs, other monoclonal antibodies were used to select potentially diagnostic antigens isolated from the *A. vasorum* adult somatic antigen for the detection of specific antibodies against *A. vasorum*. Also these ELISAs showed high sensitivity (81%) and specificity (98%), and have been validated [19] and used for seropidemiological studies [20-22]. Based on these developments, a rapid device for the serological detection of circulating antigen has been designed (Angio Detect™ Test, IDEXX Laboratories, Westbrook, Maine, USA). The test is intended for routine in-clinic diagnosis, since the rapid detection of infected dogs is a key point for initiating an indispensable and urgent therapy. The aim of this study was to evaluate this newly developed in-clinic test.

## Methods

Sera from naturally and experimentally infected dogs have been used for determination of sensitivity and specificity. In detail:

a) 39 serum samples from naturally infected dogs were used for determination of sensitivity: 28 sera from Switzerland from dogs suspected for canine angiostrongylosis and confirmed positive by microscopic identification of first stage larvae of *A. vasorum* after Baermann-Wetzel technique [7] at the Institute for Parasitology in Zurich, and 13 sera from Germany from previous lungworm studies [18,23], were also confirmed positive by larval analysis.

b) Sera from 28 dogs experimentally inoculated with a European isolate [5,18,24] and with known worm burden were used for a temporal follow up starting from samples before inoculation and samples collected 21, 35, 49, 62 (± 1, n = 28), 76 (± 1, n = 20), 91 (+1, n = 15) and 97 (n = 4) days post inoculation (dpi). Two dogs were followed-up until 286 dpi. Baermann-Wetzel results of these dogs were available (all dogs became patent within 47-55 dpi).

c) The sera of a further 10 experimentally inoculated dogs were followed up from before inoculation and before and after anthelmintic treatment. Treatment was performed between 81 and 110 dpi and consisted of the administration of imidacloprid 10 mg/kg body weight (BW)/moxidectin 2.5 mg/kg BW spot-on. This dose corresponds to 0.1 ml product (Advocate®) per kg BW. Larval counts were also available for these dogs (all dogs became patent within 47-53 dpi).

d) Cross-reactivity was tested with a total of 121 samples from dogs with proven parasitic infections. In detail, sera of dogs experimentally infected with *Toxocara canis* (n = 21) [25] or *Ancylostoma caninum* (n = 4, from the Institute of Parasitology, University of Veterinary Medicine, Hannover), of German [23] and Swiss dogs naturally infected with *Crenosoma vulpis* (n = 18) and (Czech) dogs positive for *Oslerus osleri* (n = 3) diagnosed by the presence of L1 in faeces, of Italian dogs positive for *Eucoleus aerophilus* (syn. *Capillaria aerophila*) eggs (n = 6) detected by coproscopy after flotation and confirmed by PCR-coupled sequencing [26], of dogs positive for *Dirofilaria immitis* (n = 28) diagnosed by the presence of circulating antigen (DiroCHEK®, Synbiotics, San Diego, USA) and/or microfilariae (which were characterized with the acid phosphatase stain), of dogs positive for *Dirofilaria repens* (n = 20), *Acantocheilonema reconditum* (n = 10) or *Dipetalonema dracunculoides* (n = 10), diagnosed by the presence of microfilariae which were characterized with the acid phosphatase stain and/or length measurements and/or PCR [27], were tested. Also one dog with contemporaneous presence of *A. reconditum* and *D. repens*, and two dogs diagnosed with a double infection with *A. vasorum* and *C. vulpis* were tested.

e) Finally, 10 control sera of selected dogs negative by Baermann-Wetzel analysis were tested.

**Figure 1:**
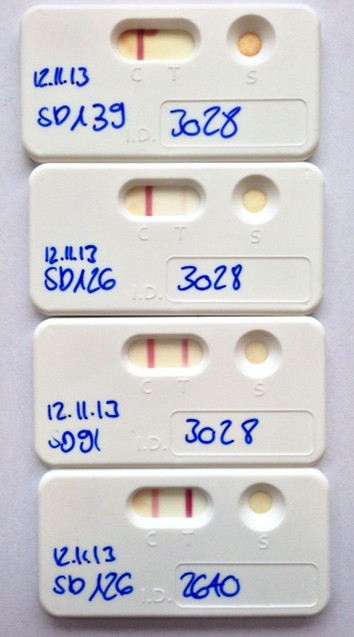
**Colour intensities in the Angio Detect™ Test.** As an example: Angio Detect™ Test results of two dogs (ID no. 3028 and 2640) inoculated with third stage larvae of *Angiostrongylus vasorum* and treated with Advocate® at 91 days after inoculation. No coloration was observed in the serum of dog 3028 48 days after treatment (study day SD 139); the same dog was "+" (slight but visible coloration) 35 days after treatment (SD = 126) and "++" (good visible coloration) 91 days after inoculation (SD = 91). Dog 2640, from which an adult parasite was detected at necropsy, was still "+++" (intensive coloration) 35 days after treatment (SD 126).

All sera were tested with the Angio Detect™ Test (IDEXX Laboratories), a lateral flow immunochromatography test which includes a positive control field. Tests were performed by experienced laboratory staff following the manufacturer’s direction and within the indicated expiry dates. Results were semiquantitatively evaluated based on the colour intensity (+ = slight but visible coloration, ++ = good visible coloration, +++ = intensive coloration, see Figure 1). Furthermore, sera were also tested with the ELISA for detection of circulating antigen of *A. vasorum*[18]. For a semiquantitative comparison with the results of the Angio Detect™ Test, optical density (OD) values were graduated into negative (< 0.159), + (0.159 - 0.350), ++ (0.351 - 0.800) and +++ (> 0.800) results.

Excel 2007 for Windows (Microsoft Corporation, Redmond, USA) was used to calculate the prevalence values, means and standard deviations (SD). Sensitivities were calculated dividing the number of seropositive animals by the total number of infected animals, while specificities were calculated dividing the number of seronegative animals by the total number of uninfected animals tested. Exact binomial 95% confidence intervals (95% C.I.) for means of binomial variables were calculated with unweighted data according to the method of Clopper and Pearson [28].

All institutional and national guidelines for the care and use of laboratory animals were followed. Experiments with dogs were carried out with facility-born animals at the experimental units of the Vetsuisse Faculty in Zurich upon approval by the Cantonal Veterinary Office of Zurich (permission numbers 25/2006, 26/2007, 10/2008, 13/2008, 185/2008).

## Results

Serological Angio Detect™ Test results obtained from dogs naturally infected with *A. vasorum* are shown in Table 1. A total of 33 out of 39 Baermann-positive dogs were positive, resulting in a sensitivity of 84.6% (95% C.I. 69.5 - 94.1%). In comparison, 37 out of 39 of the sera were also confirmed seropositive for detection of circulating antigen by ELISA (sensitivity 94.9%, 95% C.I. 82.7 – 99.3%). Two out of 6 sera which were negative with the Angio Detect™ Test were also ELISA negative, while the other 4 sera were in the lower OD category (+, OD = 0.159-0.350) of positive results, above the cut-off value.

**Table 1:** **Serological results of 39 dogs naturally infected with *Angiostrongylus vasorum *** **(as diagnosed with positive Baermann-Wetzel analysis) tested with the Angio Detect™ Test (IDEXX Laboratories) and with an ELISA for detection of circulating antigen**[18]

	**Antigen-ELISA**^**2**^				
**Angio-detect™**^**1**^	**Negative**	**+**	**++**	**+++**	**Total**
	**(< 0.159)**	**(0.159-0.350)**	**(0.351-0.800)**	**(>0.800)**	
Negative	2	4			6
+			1	3	4
++				8	8
+++				21	21
Total	2	4	1	32	39

^1^negative = no coloration, + = slight, visible coloration, ++ = good visible coloration, +++ = intensive coloration (see Figure 1).

^2^ELISA-results categorized based on optical density values (given in brackets).

The serological follow-up of dogs experimentally inoculated with *A. vasorum* showed that seropositivities detected by Angio Detect™ Test followed seropositivities detected by ELISA in time (Table 2). The earliest positive results with the Angio Detect™ Test were observed at 9 weeks post inoculation: 20 out of 28 sera were ELISA-positive, and five of them were also Angio Detect™ Test positive. At 11 weeks post inoculation, all tested 20 dogs were seropositive with the ELISA, and 10 of them were also Angio Detect™ Test positive. Thirteen weeks post inoculation (91 dpi), 15 sera were tested, and again all were ELISA-positive, and only 4 sera were Angio Detect™ Test negative: of these, 3 were tested again 97 dpi, and they all became Angio Detect™ Test positive. Dogs followed-up for up to 286 dpi remained constantly seropositive. Concerning the intensity of coloration on the test strips, intensity was by trend higher the longer the dogs were infected. In opposition, colour intensity was not correlated with the number of adult worms determined at necropsy (results not shown).

**Table 2:** **Serological results of dogs experimentally inoculated with *Angiostrongylus vasorum *** **tested with the Angio Detect™ Test (IDEXX Laboratories) and with the ELISA for detection of circulating antigen**[18]

	**No. of dogs**	**Antigen-ELISA-positive**		**Angio Detect™ Test -positive**	
**Weeks after inoculation**	**n**	**n**	**% (95% CI)**	**n**	**% (95% CI)**
0	28	0	0 (0-10.1)	0	0 (0-10.1)
3	28/9^1^	0	0 (0-10.1)	0	0 (0-28.3)
5	28/9^1^	6	21.4 (8.3-41.0)	0	0 (0-28.3)
7	28	11	39.3 (21.5-59.4)	0	0 (0-10.1)
9	28	20	71.4 (51.3-86.8)	5	17.9 (6.1-36.9)
11	20	20	100 (86.1-100)	10	50.0 (27.2-72.8)
13	15	15	100 (81.9-100)	11	73.3 (44.9-92.2)
14	8	8	100 (68.8-100)	8	100 (68.8-100)
15-41	2	2	100 (22.4-100)	2	100 (22.4-100)

Baermann-Wetzel confirmed patency in all dogs within 47-55 dpi.

^1^only 9 out of the 28 dogs were tested with the Angio Detect™ Test.

Out of 10 dogs treated at 81-110 dpi with the spot-on solution containing moxidectin/imidacloprid (Advocate®), seven became positive in the Angio Detect™ Test at 49 (1 dog), 62 (1 dog), 76 (2 dogs), 91 (2 dogs) or 104 (1 dog) dpi. They all turned negative again within 3 (3 dogs) to 7 weeks (3 dogs) after treatment, with one exception: one dog in which one worm was still detected at necropsy remained positive in the Angio Detect™ Test (see also Figure 1) and in the ELISA as well until necropsy (6 weeks after treatment), but was negative for larval excretion. Angio Detect™ Test results were confirmed by negative ELISAs within 3-9 weeks after treatment (after 3, 5, 7 and 9 weeks in 2, 3, 1, and 2 dog, respectively) and absence of larval excretion within 9-20 days after treatment.

As expected, the sera of the two dogs with simultaneous infection with *C. vulpis* and *A. vasorum* were positive. All 121 sera tested for potential cross-reactions were negative with the Angio Detect™ Test, indicating a specificity of 100% (95% C.I. 97.6 - 100%). Also the 10 selected sera with negative Baermann-Wetzel analysis were negative with the Angio Detect™ Test, indicating a specificity of 100% (95% C.I. 74.1-100%).

## Discussion

The diagnosis of canine angiostrongylosis is particularly challenging, since clinical signs may be highly variable. Based on studies with experimentally infected dogs, respiratory signs can be detected approximately seven weeks post inoculation [24], more or less simultaneously with the onset of patency and detection of circulating antigen by ELISA [18]. However, very frequently early clinical signs may not be visible and the onset of potentially fatal coagulopathies may occur [29-32], which may be associated with chronic infections [24,30,33]. Since the first description of *A. vasorum* in France in the 19^th^ century [34], endemic foci have been described in different European countries, and in the last decades an increasing number of cases have been reported from Europe [21] and from North America in the Atlantic Canadian province of Newfoundland and Labrador [35]. *A. vasorum* was detected in dogs and in foxes, with potential for expansion and establishment in other areas [36]. This increase of reported cases may be attributed to increased disease awareness from the veterinary and from the pet owner’s side as well, but also due to available new diagnostic techniques such as molecular tools [37-39] or ELISAs, contributing to increased knowledge and diagnosis of *A. vasorum*. Molecular techniques are particularly recommended for prevalence studies in intermediate mollusc hosts [40] and for confirmative diagnosis in dogs or foxes [37], while ELISAs have been used for mass-screening in large epidemiological investigations [21,22]. Additionally, as previously shown, the specific detection of circulating *A. vasorum* antigen by ELISA also represents a valid alternative for reliable diagnosis and for follow-up investigations after anthelmintic treatment [18].

Both tests, Angio Detect™ Test and the ELISA for detection of circulating antigen, are intended to diagnose an active *A. vasorum* infection, and sensitivity evaluated on naturally infected dogs of this study were 84.6% (95% C.I. 69.5 - 94.1%) and 94.9% (95% C.I. 82.7 – 99.3%), for the rapid device and the ELISA, respectively. The sensitivity of the ELISA determined in this study was absolutely comparable with the results previously obtained with sera of 23 naturally infected dogs (95.7%, 95% CI 78.1-99.9% [18]).

Other commercially available tests based on the same principle as the Angio Detect™ Test (lateral flow immunochromatography), in particular tests for the detection of circulating antigen of *D. immitis*, were frequently showing lower sensitivities compared to plate ELISA assays which involve multiple steps including signal amplification. In a study performed with sera of dogs harbouring low heartworm burdens, sensitivities of the 3 tested lateral flow immunochromatography tests varied between 52 and 71%, while three test kits based on ELISA had sensitivities between 67-71% [41]. In another study performed with dog sera collected in Argentina, two kits based on lateral flow immunochromatography and one on membrane ELISA all had a sensitivity of 76% [42]. Lower sensitivities of test kits based on lateral flow immunochromatography compared with ELISAs has also been reported for other parasites such as *Leishmania* infections in dogs, with sensitivities varying between and 53% and 64% (asymptomatic dogs), and 76% and 97% (symptomatic dogs) for test kits and ELISAs, respectively [43]. In this context, the calculated sensitivity of 84.6% of the Angio Detect™ Test can be considered as high and is additionally confirmed by detection of an *A. vasorum* infection in a treated dog which was negative for larval excretion but in which one worm was still detected at necropsy. In fact, as already mentioned, the Baermann-Wetzel technique can be false negative also after prepatency.

Four out of 6 sera of naturally infected dogs which were negative with the Angio Detect™ Test were allocated to the "low positive" category in the ELISA: possibly, these dogs would have become seropositive with the Angio Detect™ later. Differences of sensitivities between Angio Detect™ Test and ELISA for detection of *A. vasorum* infections are illustrated more in detail by the results obtained when testing experimentally infected dogs. With increasing time after inoculation, seropositivity increased for both tests: detection of circulating antigen with the ELISA started from 5 weeks after inoculation and all tested dogs were positive starting from 11 weeks after inoculation. In contrast, first seropositive results with the Angio Detect™ Test were obtained starting from 9 weeks after inoculation and were positive for all dog sera at 14 weeks after inoculation. The evaluation of the colour intensity of the test strips confirmed the delay of approximately 3-4 weeks for antigen detection by the Angio Detect™ Test and its correlation with the time after infection, independent of the worm burden detected at necropsy. However, this delay could be of minor importance for prevention of potentially deadly cases: as previously shown, despite heavy *A. vasorum* infection load and pulmonary changes, only mild haematological changes were observed in experimentally infected dogs up to 13 weeks after inoculation [24], indicating that fatal coagulopathies may occur only afterwards. At this time, sensitivity of the Angio Detect™ Test increased up to 100%, and was additionally complemented with a very high specificity of 100% (95% C.I. 97.6-100%).

Interestingly, both ELISA and Angio Detect™ Test were negative for two dogs diagnosed positive by Baermann-Wetzel technique. A tentative explanation of this result could be the formation of antigen-antibody complexes, which may inhibit detection of antigen in some canine samples, as it has been shown with *D. immitis*[44,45]. Similarly, during the evaluation of the antigen-ELISA [18], the follow-up of an experimentally infected dog harbouring 165 adult *A. vasorum* at necropsy evidenced OD values which were constantly under the cut-off value, except at 35 dpi, possibly due to relevant amounts of circulating antigens and antibodies forming antigen-antibody complexes. In order to test this hypothesis, we re-tested the serum of this latter dog and some of the sera of the study presented here, for which an adequate amount of serum was available after pretreatment with heat, as previously described [46]. One out of two sera of the naturally infected dogs negative for both, the antigen-ELISA and the Angio Detect™ Test (see Table 1), turned out positive in both tests. A further 12 sera of naturally and experimentally infected dogs with contradictory results (antigen-ELISA positive and Angio Detect™ Test negative) were retested after heat treatment: 9 of them were then also positive in the Angio Detect™ Test (data not shown). This indicates that pretreating the sera may increase the sensitivity of the test. However, there were still sera of dogs with proven experimental infections that remained negative, in opposition to the ELISA result.

Currently, canine angiostrongylosis is diagnosed with analysis of faeces by the Baermann-Wetzel method, having the disadvantage that larval migration is usually performed overnight and additionally samples are often sent to a laboratory, causing a delay in diagnosis. Considering that the Angio Detect™ Test can be directly used in veterinary practices for suspect dogs, this test represents a valid alternative for immediate diagnosis and subsequent concerted treatment of angiostrongylosis. Certainly, coproscopic methods still remain an option in suspect dogs and with negative Angio Detect™ Test results, and represent the only option for diagnosis of other lung worms such as *Crenosoma vulpis* or *Eucoleus aerophilus*.

## Conclusions

This study provided evidence of a good sensitivity and a very high specificity of the rapid device Angio Detect™ Test for detection of circulating *A. vasorum* antigen in dogs with suspected canine angiostrongylosis. As the test is conceived for in clinic-use, its use is simple and easy and can be promptly and broadly applied, representing a very useful tool to be used in veterinary practices.

## Competing interests

MS and PD were involved in the development of the Angio Detect™ Test.

## Authors’ contributions

MS participated in the design of the study, collected the samples, carried out the diagnostic assays and drafted the manuscript. KS carried out the diagnostic assays. TJN collected the samples and contributed to the draft of the manuscript. SL collected the samples and carried out the diagnostic assays. PD participated in the design of the study and the draft of the manuscript. All authors have read and approved the final manuscript.
